# Cardiovascular Disease Death Before Age 65 in 168 Countries Correlated Statistically with Biometrics, Socioeconomic Status, Tobacco, Gender, Exercise, Macronutrients, and Vitamin K

**DOI:** 10.7759/cureus.748

**Published:** 2016-08-24

**Authors:** David K Cundiff, Paul S Agutter

**Affiliations:** 1 Internal Medicine, LA County + USC Medical Center (Retired); 2 Formerly with Theoretical Medicne and Biology Group, Formerly University of Edinburgh

**Keywords:** vitamin k2, cardiovascular mortality, tobacco, diabetes, hypertension, global burden of disease, air pollution, diet, body mass index

## Abstract

**Background:**

Nutrition researchers recently recognized that deficiency of vitamin K2 (menaquinone: MK-4–MK-13) is widespread and contributes to cardiovascular disease (CVD). The deficiency of vitamin K2 or vitamin K inhibition with warfarin leads to calcium deposition in the arterial blood vessels.

**Methods:**

Using publicly available sources, we collected food commodity availability data and derived nutrient profiles including vitamin K2 for people from 168 countries. We also collected female and male cohort data on early death from CVD (ages 15–64 years), insufficient physical activity, tobacco, biometric CVD risk markers, socioeconomic risk factors for CVD, and gender. The outcome measures included (1) univariate correlations of early death from CVD with each risk factor, (2) a multiple regression-derived formula relating early death from CVD (dependent variable) to macronutrient profile, vitamin K1 and K2 and other risk factors (independent variables), (3) for each risk factor appearing in the multiple regression formula, the portion of CVD risk attributable to that factor, and (4) similar univariate and multivariate analyses of body mass index (BMI), fasting blood sugar (FBS) (simulated from diabetes prevalence), systolic blood pressure (SBP), and cholesterol/ HDL-C ratio (simulated from serum cholesterol) (dependent variables) and dietary and other risk factors (independent variables).

**Results:**

Female and male cohorts in countries that have vitamin K2 < 5µg per 2000 kcal/day per capita (n = 70) had about 2.2 times the rate of early CVD deaths as people in countries with > 24 µg/day of vitamin K2 per 2000 kcal/day (n = 72). A multiple regression-derived formula relating early death from CVD to dietary nutrients and other risk factors accounted for about 50% of the variance between cohorts in early CVD death. The attributable risks of the variables in the CVD early death formula were: too much alcohol (0.38%), too little vitamin K2 (6.95%), tobacco (6.87%), high blood pressure (9.01%), air pollution (9.15%), early childhood death (3.64%), poverty (7.66%), and male gender (6.13%).

**Conclusions:**

Worldwide dietary vitamin K2 data derived from food commodities add much understanding to the analysis of CVD risk factors and the etiology of CVD. Vitamin K2 in food products should be systematically quantified. Public health programs should be considered to increase the intake of vitamin K2-containing fermented plant foods such as sauerkraut, miso, and natto.

## Introduction

Paradoxes concerning CVD abound. Food scientists hotly dispute whether a plant-based diet or an omnivorous diet is optimal for the prevention of CVD [1]. Cardiologists debate whether the vulnerable plaque hypothesis to explain coronary artery disease (CAD) events, a foundational basis of lipid-lowering treatment, should be abandoned [2]. A recent trial showed that evacetrapib, a drug that lowers low-density lipoprotein (LDL) cholesterol, had no effect on the CVD outcomes, bringing into question the mechanism of the benefit of statins in reducing CVD events [3]. Counterintuitively, while the incidences of obesity and diabetes (i.e. risk factors for CVD) have risen during recent decades in Western countries, deaths attributed to CVD have fallen markedly [4].

Globally, about 30% of all deaths are due to CVD. About 38% of people in high-income countries die of CVD compared to 28% in low and middle-income countries [5]. However, CVD death rates among young people (< age 65) are higher in low and middle-income countries because of the shorter average lifespan. Nearly 80% of deaths in high-income countries occur among those over the age of 60 compared to 42% in low and middle-income countries [6]. These and the other CVD paradoxes call for new hypotheses that better explain the diverse and puzzling data. This paper will present data that supports the hypothesis that vitamin K2 (menaquinones: MK-4–MK-13) plays a central role in CVD etiology, epidemiology, and pathogenesis.

Vitamin K comprises a family of fat-soluble, structurally-similar compounds that function as enzymatic co-factors in the cross-linking of γ-carboxyl with ε-amino side chains in vitamin K-dependent proteins. Vitamin K-dependent proteins include not only blood coagulation proteins manufactured in the liver but also components of many extrahepatic tissues including arterial vessels and bones. Vitamin K1 (phylloquinone) comes primarily from green leafy vegetables. Vitamin K2 molecules are designated as MK-4–MK-13 according to the lengths of their isoprenyl side chains attached to a 2-methyl-1,4-naphthoquinone ring. Some vitamin K2 comes from dietary animal products without bacterial action (MK-4). Other vitamin K2 originates from bacterial action in animal and human guts and from bacterial action in fermenting plants and dairy products (MK-5–MK-13) [7]. In conjunction with vitamin D, vitamin K2 regulates the deposition of calcium, so bones and teeth receive calcium while blood vessels such as coronary arteries do not [8].

Animal trials and human observational studies have demonstrated that vitamin K2 deficiency (dietary deficiency or vitamin K inhibition by warfarin) contributes to CVD by stiffening and calcifying coronary arteries and other vessels [9]. An eight-year-long observational study involving 4,807 men and women aged 55 years and older in Rotterdam, Netherlands found that people in the lowest tertile of intake (vitamin K2 < 21.6 µg/day) had 27% more CVD mortality than people in the mid tertile (vitamin K2 = 21.6–32.7 µg/day) and 57% more than those in the upper tertile (vitamin K2 > 32.7 µg/day) [10]. As per the Multi-Ethnic Study of Atherosclerosis (MESA) in the United States, CVD incidence over 11 years of observation increased progressively as vitamin K2-dependent protein activity decreased, with event rates of 5.9 and 11.7 per 1000 person-years in the highest and lowest quartiles, respectively [11].

Biometric markers such as BMI, FBS, hemoglobin A1c, SBP, and serum cholesterol/HDL-cholesterol ratio (TC/HDL) have been correlated with CVD events in developed countries [12]. Socioeconomic risk factors such as dropping out of school, poverty, and certain occupations have also been correlated with CVD [13]. It has been found that nutritional and other stresses on infants and young children are associated with higher CVD death rates later in life. Study of infants in utero during the influenza pandemic of 1918 [14] and during the Dutch famine of 1944 [15] showed that these individuals suffered increased rates of CVD deaths in later life. Early childhood mortality (age 0–5 years) provides a reasonable index of fetal, infant, and early childhood distress (FICD) that might correlate with mortality from CVD in early adult life and middle age.

This study will use multiple regression analysis of female and male cohort data worldwide to relate early death from CVD (dependent variable) with major CVD risk factors (independent variables) to determine the attributable risks for each of these factors. For conditions associated with CVD (i.e. obesity, diabetes, hypertension, and increased TC/HDL), similar multiple regression analysis-derived formulae will be used to determine the attributable risks.

## Materials and methods

For the univariate and multiple regression analyses presented in this paper, we correlated CVD risk factors (diet, tobacco, biometrics (BMI, SBP, TC/HDL, FBS), socioeconomic factors (poverty, early childhood death (0–5 years old), years of education [16], air pollution [17]), and gender) with CVD-related outcomes (early CVD death (ages 15–64 years), BMI, SBP, TC/HDL, and FBS). 

In late 2015, the United States Department of Agriculture (USDA) National Nutrient Database Release 28 [18] for the first time contained data on dietary menaquinone-4 (MK-4), a major component of vitamin K2. Menaquinone data (MK-4–MK-13) have been published in European and Japanese studies and compiled into a database [19]. We used these two data sources to derive average vitamin K1 and K2 levels in 20 plant- and animal-based food commodities.

For this study, data from countries around the world was provided by the World Health Organization (WHO) [20], the Food and Agriculture Organization (FAO) [21], the Institute for Health Metrics and Evaluation (IHME) [16, 22-23], and the International Diabetes Federation (IDF) [24]. Table 1 shows the categories of datasets available from these sources for female and male cohorts.

**Table 1:** CVD-related data sources for analysis

CVD-related Outcomes and Risk Factors	Data Source	Countries	Cohorts
Death from CVD, ages 15-64 years [23]	IHME	185	370
Food commodities available [21]	FAO	175	350
Diabetes prevalence, 40-49 year olds [24]	IDF	198	396
Serum cholesterol (age standardized) [20]	WHO	187	373
Mean BMI, ages>15 years (BMI) [20]	WHO	188	376
Mean systolic blood pressure (SBP), ages >18 [20]	WHO	188	376
Insufficient physical activity, ages >18 [20]	WHO	142	284
Tobacco use, ages >15 [20]	WHO	182	363
Alcohol consumption, ages >15 [20]	WHO	187	374
Mean percapita gross domestic product (GDP ) [25]	WHO	201	402
Child mortality (0-5 years old) [20]	WHO	173	346
Air pollution (deaths caused by air pollution/100k) [22]	IHME	185	370
Education (years of schooling) [16]	IHME	173	346

Utilizing the formulae and data in this paper to later create a CVD risk modeling app will require that all of the variables analyzed must correspond to CVD risk factors of individuals. While the WHO supplies worldwide data on FBS (mmol/L), the cohort data is rounded off to two digits and has a relatively narrow range (4.7–6.9 mmol/L). On the other hand, the IDF data on diabetes prevalence for female and male cohorts 40–49 years old are calculated to three digital places that range from 0.50% to 51.8%. Consequently, the IDF diabetes prevalence data was modified to represent simulated FBS data by first adjusting the standard deviation (SD) of the diabetes prevalence dataset to equal the SD of WHO FBS cohorts from 2009. Then the mean was adjusted to equal the mean of the FBS dataset as shown:

Simulated FBS = diabetes prevalence 2015 (IDF) * 0.0332012314 + 4.89865

If data on early mortality from CVD and food commodity availability was present, we included the country in the analysis. The FAO provided data on plant- and animal-based food commodities as kilocalories available per capita/day (kcal/day). Table 2* *shows the 20 plant-based and animal-based commodities we evaluated.

**Table 2:** Plant-based and animal-based food commodities

#	Food Commodities
1	Cereals and grains
2	Starchy vegetables
3	Sweets and sugar added sweets (many containing dairy products)
4	Pulses (legumes)
5	Tree nuts
6	Vegetable oils
7	Oil extraction by-products
8	Vegetables
9	Fruits and fruit juices
10	Beef
11	Butter
12	Cheese
13	Cream
14	Eggs
15	Milk products
16	Mutton
17	Pork
18	Poultry
19	Fish and sea food
20	Offal

For this analysis, we used WHO data on alcohol consumption (g/day) by gender, rather than the alcohol availability data by country from the FAO.

These food commodities accounted for 98.6% of the total kcal/day available on average for each country. We used the averages of the food commodity data for the years 1991, 2001, and 2011. 

We utilized data about vitamin K2 levels in plant and animal products from European and Japanese studies and averaged the European MK-4 levels together with the USDA MK-4 findings (MK-4 data unavailable for mutton). We then included the European MK-5–MK-13 data by food commodity to generate complete vitamin K2 content estimates for 17 of the 20 food commodities (MK-5–MK-13 data unavailable for mutton, sugars and sweets, and fish).

This study used the USDA National Nutrient Database to derive average values for the 12 nutrients of interest for each of these 20 food commodities excluding alcohol. These nutrients are included in Table 3.

**Table 3:** Macronutrients and vitamin K forms utilized

	Nutrients
1	Protein (% of kcal/day and g)
2	Carbohydrates (% of kcal/day and g)
3	Dietary fiber (g/1000 kcal/day and g)
4	Total fat (% of kcal/day and g)
5	Saturated fatty acids (% of kcal/day and g)
6	Monounsaturated fatty acids (% of kcal/day and g)
7	Polyunsaturated fatty acids (PUFA) (% of kcal/day and g)
8	Trans fatty acids (% of kcal/day and g)
9	Vitamin K1 (µg/1000 kcal/day and µg)
10	MK-4 (µg/1000 kcal/day and µg)
11	MK-5-13 (µg/1000 kcal/day and µg)
12	Vitamin K2 (µg/1000 kcal/day and µg)

For each country, we used the mean availability of each food commodity (kcal/day) from the FAO in conjunction with the USDA nutrient database to quantify the macronutrient profile, vitamin K1, and K2 (MK-4–MK-13). For each of the 20 food categories listed above, we found all available food items from the USDA nutrient database to create a reference profile of the average quantity per 100 g portion for each nutrient under study—i.e. energy (kcal/day), macronutrients (g), vitamin K1 (µg), and vitamin K2 (µg). The reference nutrient profile for each of the 20 food components is shown in Supplementary Table 3. The steps we used to determine the nutrient profile for each country are detailed in Table 4.

**Table 4:** Steps in converting food components to nutrient profiles

Step #	Steps in Forming Nutrient Profiles for Countries
1	For each country we determined the kcal/day available in each of the 20 food categories.
2	For each food category, we used the USDA nutrient database to determine the average nutrient profile in kcal per 100 g portion of foods in that category (i.e. protein (g), carbohydrates (g), dietary fiber (g), fat (g), vitamin K2 µg, etc.)
3	For instance, 100 g of pork from the USDA nutrient database averaged 162 kcal and had 18.6 g of protein, 0.30 g of carbohydrate, 0.0 g of dietary fiber, 9.2 g of total fat, 0.06 µg of vitamin K2, etc.
4	For each country, we then divided the total kcal available in each food group by the kcal per 100 g of that food category. This gave 20 multipliers corresponding to the 20 food commodities. For example, for country A, 100 kcal/day was available as pork out of 3000 total kcal/day available on average. From the USDA nutrient database, 100 g of pork contained 162 kcal; so the multiplier for pork would be 100 kcal/day of pork/162 kcal (in 100 g of pork) = 0.6173).
5	For each country, we then multiplied the kcal of each food commodity by the corresponding unique multiplier for each component of the nutrient profile. For example, for country A, the protein from pork was 18.6 g (in 100 g of pork) * 0.6173 = 11.48 g/day of protein from pork). (The symbol * denotes multiplication.)
6	Finally, we derived the values for each nutrient of interest in g or µg by summing the amount from each of the 20 food groups.

The following formula describes the nutrient profiles from the food commodity data using each of the 20 food groups to derive the 12 nutrients of interest (N1–N12) for each country:

$Food{\hspace {.1cm}} groups{\hspace {.1cm}} to{\hspace {.1cm}} nutrients {\hspace {.1cm}}transformations = \sum _{1}^{20} N1...N12$

The kcal/day for each macronutrient was converted into g/day by the appropriate conversion factor (protein: 4 kcal/g, carbohydrates: 4 kcal/g, fats: 9 kcal/g). We expressed dietary fiber both as g/1000 kcal and as g. For vitamin K1 and K2, we derived the µg/day per 1000 kcal/day from the average contents of these vitamins in the 20 food groups.

For alcohol, we used WHO data on consumption per day (g) by gender instead of the kcal/day availability per capita data from the FAO.

For the nutrient profiles, we estimated the kcal/day available by gender and consequently the food group availability by gender based on National Health and Nutrition Examination Survey data, which reported that males consume about 50% more calories than females [26].

For the analyses of impacts of food groups, we correlated early CVD death with kcal per 1000 total kcal available of each food group (e.g. if for beef = 100 kcal/day out of the 3000 total kcal/day on average was available, then beef = 33.3 kcals/1000 kcal). For the analyses of macronutrients, we determined the percentage of kcal/day available that came from each macronutrient with caloric value (protein, carbs, fat, and alcohol). For dietary fiber and vitamins K1 and K2, we determined the g/day per 1000 kcal (fiber) and µg per 1000 kcal (K1 and K2). Consequently, the impacts of food available from different food groups and availability of different nutrients were the same for both genders despite the higher caloric intake by males.

The WHO assessed the physical activity of females and males in countries worldwide with the variable “insufficient physical activity” (WHO 0%–100% scale). According to the WHO, “adults aged 18–64 should do at least 150 minutes of moderate-intensity aerobic physical activity throughout the week or do at least 75 minutes of vigorous-intensity aerobic physical activity throughout the week or an equivalent combination of moderate- and vigorous-intensity activity” [19]. The WHO defined “insufficient physical activity” as less than this recommended level of physical activity. For the 77 cohorts from 39 countries that lacked insufficient physical activity data but had records of early death from CVD, estimates based on multiple regression modeling were imputed using the insufficient physical activity as the dependent variable and selected food commodities, biometric indices, socioeconomic risk factors and gender as independent variables.

For future use of the physical activity component of this analysis in an app for people to quantify the effect of exercise on their CVD risk, it was necessary to convert 'insufficient physical activity' (yes or no: WHO 0%–100% scale) to "exercise" with more gradations of intensity of physical activity. In a previous article on diet and exercise related to BMI, DKC utilized WHO data including 'insufficient physical activity' and harmonized it with Diabetes Control and Complications Trial (DCCT) data which utilized a 1-4 DCCT exercise scale [27]. The scale was the following: sedentary (less than 30 minutes walking/day) = 1, mild exercise (30 minutes walking/day) = 2, moderate exercise (60 minutes brisk walking/day or equivalent) = 3, and vigorous exercise (90 minutes running/day or equivalent) = 4. The relationship between exercise (exer: DCCT) and insufficient physical activity (IPA: WHO) is "exer = 2.2580 + (24.73271–IPA)/33.333." The derivation of the transformation of WHO insufficient physical activity into DCCT exercise is below.

\begin{align*}IPA&=insufficient\hspace{.1 cm} physical\hspace{.1 cm} activity\hspace{.1 cm} (WHO)\\ IPA\hspace{.1 cm} range&=100\%-0\%\\ Exer&=exercise\hspace{.1 cm} (DCCT)\\Exer \hspace{.1 cm}range&=1-4\\IPA\hspace{.1 cm} range/Exer\hspace{.1 cm} range&= -33.333\\ IPA\hspace{.1 cm} mean\hspace{.1 cm} of\hspace{.1 cm} 336 \hspace{.1 cm}cohorts&=24.733\\Mean\hspace{.1 cm} sufficient \hspace{.1 cm}PE&=75.267\hspace{.1 cm} (100 - 24.733) \\Mean\hspace{.1 cm} IPA\hspace{.1 cm} on\hspace{.1 cm} the\hspace{.1 cm} DCCT\hspace{.1 cm} scale&=2.2580\hspace{.1 cm} (75.267/33.333) \\Exer&=2.2580 (mean \hspace{.1 cm}exer)+ (24.733\hspace{.1 cm} (mean\hspace{.1 cm} IPA) - IPA)/\hspace{.1 cm}33.333) \end{align*}

Due to the formula used to derive DCCT “exercise” from WHO “insufficient physical activity,” these variables were negatively correlated (r = −1.0).

For countries that had data on early death from CVD, the 20 food commodities, and alcohol, the WHO provided data on tobacco use (use of tobacco in female and male cohorts) (theoretically ranging from 0 = no tobacco use to 1 = universal tobacco use) for 325 cohorts [20], childhood mortality (deaths of children 0–5 years old/year/100,000 population) for 318 cohorts [20], and years of education for 318 cohorts [16]. As with the missing physical activity data, we inferred tobacco use, child mortality, and years of education estimates for multiple regression formulae. 

Since serum cholesterol is much less discriminating as a risk factor for CVD than is cholesterol/HDL-C ratio (TC/HDL), DKC previously simulated the TC/HDL from the serum cholesterol based on harmonizing the statistical cholesterol data from the WHO with data on TC/HDL from the DCCT. An article detailing the relationship of diet and exercise and tobacco use on TC/HDL in the DCCT database and the same variables on the simulated TC/HDL from WHO/FAO data is available on DKC’s website [28]. 

This simulation assumes that increases in serum cholesterol correspond to nonlinear increases in TC/HDL in a way that can be modeled by the means and standard deviations of the cholesterols, HDL-Cs and TC/HDLs in the DCCT database. Table 5 describes the steps that were undertaken to simulate a TC/HDL variable from cholesterol in the WHO database by harmonizing with TC/HDL from the DCCT. 

**Table 5:** Steps used to simulate TC/HDL for WHO cohorts by statistically harmonizing WHO and DCCT cholesterol data

Steps	
Step #1	Since the WHO database reported mean serum cholesterol in mmol/L, cholesterol (mmol/L) was converted to cholesterol (mg/dl) in the WHO database (i.e. multiplying cholesterol mmol/L by 38.61).
Step #2	The DCCT’s cholesterols (means and standard deviations (SDs)), HDL-C levels (means, SDs), and TC/HDLs (means, SDs) were determined by gender. These means and SDs from the DCCT database were used as the reference from which to match the means and SDs of the same variables from the WHO database. This allowed for the simulation of TC/HDL from the serum cholesterol in the WHO database.
Step #3	The SDs of the distributions of serum cholesterols of the WHO cohorts by gender were adjusted to match the SDs of the DCCT subjects by gender by multiplying by the appropriate factors (e.g. WHO simulated serum cholesterol of country A’s female cohort = mean cholesterol of country A’s female cohort + ((mean cholesterol of country A’s female cohort – 183.80234 (mean cholesterol of all female cohorts)) * SD adjustment multiplying factor (i.e. the SD adjustment factor that will harmonize the WHO female cohort cholesterol SD with the DCCT female cholesterol SD)).
Step #4	A simulated WHO HDL-C variable was created that inversely correlated with WHO serum cholesterol and had the same SD by gender as in the DCCT database. For example, simulated HDL-C for country A’s female cohort = mean cholesterol of country A’s female cohort * 0.3039 (0.3039=mean HDL-C /cholesterol for females in DCCT database) – (mean cholesterol of country A’s female cohort – 183.80 (183.80 = mean WHO female cholesterol) * 0.517 (0.517 = multiplying factor calculated to make the SD of the WHO female cohorts’ simulated HDL-Cs match the actual SD of the HDL-Cs of the DCCT females.
Step #5	Finally, the SD by gender of the WHO variable “simulated TC/HDL” was adjusted by a multiplying factor calculated to match the SD of the WHO database TC/HDL to the SD of the DCCT TC/HDL variable.

### Statistical methods

Pearson correlation coefficient analysis determined the positive or negative associations of early death from CVD with the dietary food groups and other risk factors for CVD.

We also correlated macro- and micro-nutrient intake with CVD-associated outcomes. In addition to including carbohydrates (% of kcal), dietary fiber (g/1000 kcal), total fat (% of kcal), saturated fat (% of kcal), monounsaturated fat (% of kcal), and polyunsaturated fatty acids (PUFA) (% of kcal), the ratios of total carbohydrates (g)/dietary fiber (g) and total fat (g)/PUFA (g) were included as variables. The ratio of total carbohydrates/dietary fiber gives an index of how high the diet is in refined carbohydrates (e.g. sugars and refined flours). The total fat/PUFA ratio gave an index of the proportion of dietary animal products (high ratio) versus plant products (low ratio). 

To assess the interaction of early death from CVD (dependent variable) with food commodities, macronutrients, vitamin K1, vitamin K2, physical activity, tobacco use, biometric indices, the prevalence of diabetes, socioeconomic risks, and gender (independent variables), a multiple regression analysis was deployed using the non-experimental regression method. To maximize the inclusion of as many risk factors as possible, the multiple regression analysis was conducted in stages, beginning with nutritional variables. In determining the variables to include in the formulae generated by the multiple regression analyses, we set the statistical threshold for a variable to enter and to remain in the formula at r < 0.25. This low threshold assured that all possibly important variables would be included.

The nutritional variables were analyzed by multiple regression in two stages, beginning with the percentage of kcal for macronutrients, g/1000 kcal for dietary fiber and µg/1000 kcal for vitamin K. In the second stage analysis, the nutrient variables were converted to g/µg (g for macronutrients and µg for vitamin K1 and K2) * parameter estimate/total kcal available. The third stage and subsequent analysis stages added the non-dietary risk factors to the second stage dietary ones.

In some cases, to maximize the variables included in the multiple regression analyses, we began with selected risk factors that had weaker univariate associations with early death from CVD (i.e. r = 0.15 to r = 0.30) and proceeded to risk factors with stronger univariate correlations (i.e. r = 0.30 to r = 0.80) in later stages. If a risk factor correlated directly with early death from CVD in the univariate analysis and indirectly in the multiple regression-derivation formulae, or vice versa, it was excluded from the risk modeling formula.

By multiplying each significant variable in the multiple regression by its parameter estimate and then summing the contributions of each variable, these formulae quantified the relationship between early death from CVD and the interaction of dietary and other CVD risk factors.

We derived the percentages of risk of early death from CVD attributable to individual risk factors in the multiple regression-derived formulae using the steps in Table 6:

**Table 6:** Steps in determining the attributable risks of significant risk factors

Step #	Procedures
#1	For the multiple regression-derived formula, determine the variance (i.e. R^2^).
#2	Determine the individual variances of each of the risk factors in the formula (e.g. individual R^2^s for vitamin K2, animal products, tobacco use, early childhood mortality, etc.)
#3	Total the individual R^2^s for the risk factors appearing in the formula.
#4	Divide the R^2^ of the multiple regression-derived formula by the sum of the R^2^s of the individual risk factors to generate a multiplier.
#5	Apply the multiplier to the R^2^s of the individual risk factors to determine the portion of the overall R^2^ attributable to each risk factor.
#6	To convert from R^2^ to percentage of attributable risk, multiply by 100.

We used SAS statistical software 9.1 (SAS Institute, Cary, NC) for the data analysis.

## Results

Vitamin K2 was inversely correlated with early CVD death (r = –0.41, *P* < 0.0001) to the same degree that tobacco use was positively correlated (r = 0.41, *P* < 0.0001). Using 2000 kcal/day as an average dietary intake, cohorts in countries with vitamin K2 < 5 µg/2000 kcal/day (n = 70) had about 2.2 times the rate of early CVD deaths as cohorts in countries with vitamin K2 > 24 µg/2000 kcal/day (i.e. early CVD death/100,000/year = 1729, 90% CI: 884–2797 versus 779, 90% CI: 181–2150 (n = 72)). Data on dietary vitamin K2 came exclusively from animal-based products (meat, dairy, and eggs). No data was available on vitamin K2 from fermented plant materials (e.g. sauerkraut) from countries worldwide.

Table 7 shows the correlations of food commodities with early CVD death and with vitamin K2. Table 8 shows macronutrients and vitamins K1 and K2 correlated with early CVD death and with vitamin K2. For non-dietary risk factors, Table 9 shows the correlations with early CVD death and with vitamin K2. In all these three tables, almost all the food commodity groups, nutrients, or non-dietary variables that correlated inversely with early CVD death correlated directly with vitamin K2 and vice versa. 

**Table 7:** Correlations of early death from CVD (age 15-64 years) and vitamin K2 with plant-based and animal-based food commodities (n=336, female and male cohorts from 168 countries)

Food Commodities	Kcal/per 1000 kcal/day	SD	Early CVD Death r (P)	Vitamin K2 r (P)
Butter	8.16	10.16	–0.20 (0.0003)	0.59 (<0.0001)
Cheese	12.42	15.7	–0.40 (<0.0001)	0.87 (<0.0001)
Cream	1.58	3.81	–0.26 (<0.0001)	0.63 (<0.0001)
Milk	58.38	39.33	–0.23 (<0.0001)	0.71 (<0.0001)
Eggs	8.10	5.8	–0.38 (<0.0001)	0.67 (<0.0001)
Beef	18.86	15.42	–0.02 (0.73)	0.31 (<0.0001)
Mutton	7.38	14.62	0.17 (0.0024)	0.04 (0.42)
Pork	25.98	27.28	–0.26 (<0.0001)	0.62 (<0.0001)
Poultry	19.60	18.3	–0.24 (<0.0001)	0.65 (<0.0001)
Fish	12.16	13.74	–0.02 (0.70)	0.13 (0.0148)
Offal	2.99	2.02	–0.09 (0.10)	0.39 (<0.0001)
Animal products overall	180.8	97.28	–0.29 (<0.0001)	0.89 (<0.0001)
Cereals and grains	426.9	144.6	0.31 (<0.0001)	–0.67 (<0.0001)
Starchy vegetables	69.43	85.59	0.18 (0.0011)	–0.38 (<0.0001)
Sugar and sweets	101.1	47.51	–0.28 (<0.0001)	0.57 (<0.0001)
Pulses	22.26	21.09	–0.06 (0.26)	–0.42 (<0.0001)
Tree nuts	4.33	4.88	–0.19 (0.0004)	0.24 (<0.0001)
Oil crop byproducts	22.65	35.73	0.25 (<0.0001)	–0.19 (0.0002)
Vegetable oils (e.g. soy)	92.93	39.57	–0.31 (<0.0001)	0.30 (<0.0001)
Vegetables/1000 kcals/day	19.94	11.37	–0.12 (0.0254)	0.25 (<0.0001)

**Table 8:** Correlations of early death from CVD (age 15-64 years) and vitamin K2 with macronutrients and vitamin K1 (n=336, female and male cohorts from 168 countries) * unless otherwise indicated

Macronutrients and Other Variables	% of Kcal in diet*	SD	Early CVD Death r (P)	Vitamin K2 (µg/day/per 1000 kcal/day r (P)
Protein	13.81	1.99	–0.25 (<0.0001)	0.79 (<0.0001)
Carbohydrate	58.10	8.26	0.35 (<0.0001)	–0.85 (<0.0001)
Dietary fiber g/1000 kcals	12.02	2.62	0.26 (<0.0001)	–0.77 (<0.0001)
Carbohydrates/fiber ratio	12.3	1.26	–0.08 (0.15)	0.41 (<0.0001)
Total fat	26.96	6.2	–0.30 (<0.0001)	0.69 (<0.0001)
SFA	8.27	2.71	–0.33 (<0.0001)	0.89 (<0.0001)
MUFA	10.11	2.52	–0.31 (<0.0001)	0.63 (<0.0001)
PUFA	7.13	1.74	–0.13 (0.0197)	0.13 (0.0196)
Trans fatty acids	0.33	0.13	–0.23 (<0.0001)	0.78 (<0.0001)
Total fat/PUFA ratio	3.84	0.67	–0.15 (0.0058)	0.71 (<0.0001)
Alcohol (g/day consumed*)	6.56	5.81	0.10 (0.08)	0.40 (<0.0001)
Vitamin K1 µg/1000 kcals	64.12	21.23	–0.04 (0.45)	–0.02 (0.97)
MK-4 µg/1000 kcals µg/1000 kcals	5.26	3.23	–0.39 (<0.0001)	0.96 (<0.0001)
MK-5–MK-13 µg/1000 kcals	2.09	2.03	–0.38 (<0.0001)	0.89 (<0.0001)
Vitamin K2 µg/1000 kcals	7.35	4.91	–0.41 (<0.0001)	-

**Table 9:** Correlations of early death from CVD and vitamin K2 with risk factors other than diet (n=336, female and male cohorts from 168 countries)

CVD Early Death Risk Factors	Mean	SD	Early Death From CVD r (P)	Vitamin K2 / 1000 kcal r (P)
Exercise (DCCT 1-4 scale)	2.24	0.373	0.35 (<0.0001)	–0.30 (<0.0001)
Tobacco (users/total)	0.206	0.16	0.41 (<0.0001)	0.15 (0.0055)
BMI (kg/m^2^)	25.6	2.3	–0.17 (0.0020)	0.54 (<0.0001)
TC/HDL ratio	3.63	0.91	–0.25 (<0.0001)	0.78 (<0.0001)
SBP (mmHg)	126.5	5.09	0.47 (<0.0001)	–0.30 (<0.0001)
FBS (mmol/L)	5.48	3.33	–0.06 (0.24)	0.47 (<0.0001)
Gender (female=1, male=0)	0.5	-	–0.39 (<0.0001)	-
GDP (USD)	11,077	16,455	–0.43 (<0.0001)	0.72 (<0.0001)
Early childhood mortality (ages 0-5)	40.6	43.4	0.30 (<0.0001)	–0.71 (<0.0001)
Education (years in school)	8.01	3.54	–0.20 (<0.0001)	0.76 (<0.0001)
Air Pollution (deaths/year/100,000)	53	31.4	0.47 (<0.0001)	–0.45 (<0.0001)

The multiple regression-derived CVD risk factor formula using the analysis techniques described in the Methods is:

\begin{align*} a &= alcohol \hspace{.1 cm}(\frac{g\hspace{.1 cm}consumed} {day})\\ K2 &= vitamin\hspace{.1 cm}K2\hspace{.1 cm} (\frac{\mu g}{day})\\ kcal &= kilocalories\hspace{.1 cm}available\hspace{.1 cm}per\hspace{.1 cm}day\\ t &= tobacco\hspace{.1 cm}(yes=1, no=0)\\GDP &=percapita\hspace{.1 cm} gross\hspace{.1 cm}domestic\hspace{.1 cm}product\hspace{.1 cm}(USD)\\ FICD&= fetal,\hspace{.1 cm}infant,\hspace{.1 cm}and\hspace{.1 cm}childhood\hspace{.1 cm}distress\hspace{.1 cm} (surrogate\hspace{.1 cm} for\hspace{.1 cm} early \hspace{.1 cm}childhood \hspace{.1 cm}mortality)\\ p &= Air \hspace{.1 cm}pollution \hspace{.1 cm}caused \hspace{.1 cm}deaths\hspace{.1 cm}per\hspace{.1 cm}100,000\hspace{.1 cm}per\hspace{.1 cm}year\\ SBP &= systolic\hspace{.1 cm} blood\hspace{.1 cm} pressure \hspace{.1 cm} (mm \hspace{.1 cm}Hg)\\ gender& = sex (female=1, male=0)\\ E &= Early\hspace{.1 cm}death\hspace{.1 cm}by\hspace{.1 cm}CVD \hspace{.1 cm}(ages\hspace{.1 cm}15-64\hspace{.1 cm} years)\end{align*}

\begin{align*}E &=0.66956 (0.33267 (56.216 a-114,472 (\frac{\ K2}{kcal})) +2255.8 t-0.01437 GDP+4.0646 FICD) \\&\quad +7.7398 p+25.140 SBP-171.04gender \end{align*}

(n = 336 cohorts, R^2 ^= 0.50);

Table 10 shows the correlations of plant- and animal-based food commodities with BMI, FBS, SBP, and TC/HDL. Table 11 shows the nutrient profiles related to BMI, FBS, SBP, and TC/HDL, and Table 12 presents the univariate correlations between these variables and the non-dietary risk factors.

**Table 10:** Correlations of plant-based and animal-based food commodities with BMI, FBS, SBP, and TC/HDL (n=336, female and male cohorts from 168 countries)

Food Commodities	BMI r (P)	FBS r (P)	SBP r (P)	TC/HDL r (P)
Butter	0.27 (<0.0001)	0.21 (<0.0001)	–0.19 (<0.0001)	0.58 (<0.0001)
Cheese	0.33 (<0.0001)	0.24 (<0.0001)	–0.26 (<0.0001)	0.72 (<0.0001)
Cream	0.12 (0.0302)	0.07 (0.23)	–0.10 (0.08)	0.53 (<0.0001)
Milk	0.41 (<0.0001)	0.30 (<0.0001)	–0.09 (0.11)	0.59 (<0.0001)
Eggs	0.35 (<0.0001)	0.39 (<0.0001)	–0.31 (<0.0001)	0.70 (<0.0001)
Beef	–0.02 (0.73)	–.06 (0.28)	–0.09 (0.10)	0.25 (<0.0001)
Mutton	0.18 (0.0011)	0.01 (0.81)	–0.00 (0.98)	0.11 (0.0481)
Pork	0.14 (0.0098)	0.16 (0.0026)	–0.17 (0.0013)	0.57 (<0.0001)
Poultry	0.59 (<0.0001)	0.58 (<0.0001)	–0.25 (<0.0001)	0.37 (<0.0001)
Fish	0.08 (0.12)	0.14 (0.0106)	–0.14 (0.0087)	0.20 (0.0003)
Offal	0.15 (0.0058)	0.06 (0.27)	–0.03 (0.60)	0.37 (<0.0001)
Animal products	0.49 (<0.0001)	0.39 (<0.0001)	–0.23 (<0.0001)	0.77 (<0.0001)
Cereals/grains	–0.43 (<0.0001)	–0.30 (<0.0001)	0.13 (0.0146)	–0.54 (<0.0001)
Starchy vegetables	–0.33 (0.0011)	–0.37 (<0.0001)	0.25 (<0.0001)	–0.37 (<0.0001)
Sugar and sweets	0.60 (<0.0001)	0.53 (<0.0001)	–0.23 (<0.0001)	0.47 (<0.0001)
Pulses	–0.33 (<0.0001)	–0.20 (0.0003)	0.13 (0.0200)	–0.45 (<0.0001)
Tree nuts	0.16 (0.0036)	0.19 (0.0010)	–0.20 (0.0002)	0.30 (<0.0001)
Oil by-products	0.02 (0.71)	0.02 (0.69)	0.08 (0.13)	–0.20 (<0.0001)
Vegetable oils	0.21 (<0.0001)	0.17 (0.0017)	–0.14 (0.0104)	0.25 (<0.0001)
Vegetables	0.23 (<0.0001)	0.31 (<0.0001)	–0.16 (0.0032)	0.33 (<0.0001)
Fruits/fruit juices	0.15 (0.0077)	0.12 (0.336)	–0.03 (0.61)	0.01 (0.91)
Plant products	–0.49 (<0.0001)	–0.37 (<0.0001)	0.24 (<0.0001)	–0.77 (<0.0001)

**Table 11:** Correlations of nutrients available with BMI, FBS, SBP, and TC/HDL (n=336, female and male cohorts from 168 countries)

Macronutrients and Other Variables	BMI r (P)	FBS r (P)	SBP r (P)	TC/HDL r (P)
Protein % kcal	0.38 (<0.0001)	0.36 (<0.0001)	–0.26 (<0.0001)	0.66 (<0.0001)
Carbohydrate % kcal	–0.52 (<0.0001)	–0.41 (<0.0001)	0.27 (<0.0001)	–0.73 (<0.0001)
Dietary fiber g/1000 kcal	–0.50 (<0.0001)	–0.40 (<0.0001)	0.27 (<0.0001)	–0.68 (<0.0001)
Carbohydrates/fiber ratio	0.25 (<0.0001)	0.17 (0.0016)	–0.16 (0.0031)	0.38 (<0.0001)
Total fat % kcal	0.51 (<0.0001)	0.42 (<0.0001)	–0.23 (<0.0001)	0.59 (<0.0001)
SFA % kcal	0.57 (<0.0001)	0.44 (<0.0001)	–0.25 (<0.0001)	0.78 (<0.0001)
MUFA % kcal	0.48 (<0.0001)	0.40 (<0.0001)	–0.23 (<0.0001)	0.55 (<0.0001)
PUFA % kcal	0.25 (<0.0001)	0.25 (<0.0001)	–0.11 (0.0497)	0.12 (0.0366)
Trans fatty acids % kcal	0.49 (<0.0001)	0.29 (<0.0001)	–0.19 (0.0005)	0.67 (<0.0001)
Total fat/PUFA ratio	0.37 (<0.0001)	0.20 (0.0002)	–0.11 (0.0537)	0.61 (<0.0001)
Alcohol (g/day consumed)	0.09 (0.0958)	0.02 (0.73)	0.32 (<0.0001)	0.48 (<0.0001)
Vitamin K1 µg/1000 kcal	0.04 (0.04)	0.09 (0.12)	–0.01 (0.93)	0.08 (0.16)
MK-4 µg/1000 kcal	0.59 (<0.0001)	0.55 (<0.0001)	–0.31 (<0.0001)	0.72 (<0.0001)
MK-5–MK-13 µg/1000 kcal	0.33 (<0.0001)	0.26 (<0.0001)	–0.23 (<0.0001)	0.74 (<0.0001)
Vitamin K2 µg/1000 kcal	0.54 (<0.0001)	0.47 (<0.0001)	–0.30 (<0.0001)	0.78 (<0.0001)

**Table 12:** Correlations of non-dietary CVD risk factors with BMI, FBS, SBP, and TC/HDL (n=336, female and male cohorts from 168 countries)

CVD Early Death Risk Factors	BMI r (P)	FBS r (P)	SBP r (P)	TC/HDL r (P)
Exercise (DCCT 1-4 scale)	–0.51 (<0.0001)	–0.48 (<0.0001)	0.41 (<0.0001)	–0.20 (<0.0001)
Tobacco (yes=1, no=0)	0.03 (0.96)	0.20 (0.0003)	0.24 (<0.0001)	0.38 (0.0230)
BMI (kg/m2)	-	0.66 (0.0020)	–0.19 (0.0020)	0.45 (<0.0001)
FBS mmol/L	0.66 (<0.0001)	-	–0.06 (0.24)	0.48 (<0.0001)
SBP (mmHg)	–0.19 (0.0006)	–0.27 (<0.0001)	-	–0.19 (<0.0001)
TC/HDL	0.56 (<0.0001)	0.52 (<0.0001)	–0.41 (<0.0001)	-
Gender (female=1, male=0)	0.15 (0.0074)	–0.01 (0.84)	–0.51 (<0.0001)	0.26 (0.0068)
GDP (USD)	0.27 (<0.0001)	0.23 (<0.0001)	–0.37 (<0.0001)	0.72 (<0.0001)
Fetal, infant, childhood distress (surrogate for early childhood mortality)	–0.62 (<0.0001)	–0.65 (<0.0001)	0.37 (<0.0001)	–0.75 (<0.0001)
Education (years of school)	0.53 (<0.0001)	0.46 (<0.0001)	–0.23 (<0.0001)	0.73 (<0.0001)
Air Pollution (deaths/year/ 100,000)	–0.47 (<0.0001)	–0.41 (<0.0001)	0.26 (<0.0001)	–0.40 (<0.0001)

In order to derive formulas for BMI, FBS, SBP, and TC/HDL, we used the same multiple regression approach that was used to relate CVD early deaths to risk factors. Nutrients as dietary variables were used instead of food groups so as to be more applicable for later utilization in an online app.

BMI formula:

\begin{align*} a &= alcohol\hspace{.1 cm}\frac{g\hspace{.1 cm} per\hspace{.1 cm} day} {percapita}\hspace{.1 cm}by\hspace{.1 cm}gender\\ prot &= protein(g\hspace{.1 cm}per\hspace{.1 cm}day\hspace{.1 cm}available)\\ carbs &= carbohydrates\hspace{.1 cm} (g\hspace{.1 cm}per\hspace{.1 cm}day\hspace{.1 cm}available) \\ fiber &= dietary\hspace{.1 cm} fiber\hspace{.1 cm} (g\hspace{.1 cm}per\hspace{.1 cm}day\hspace{.1 cm}available)\\ TF &= total fat\hspace{.1 cm} (g\hspace{.1 cm}per\hspace{.1 cm}day\hspace{.1 cm}available) \\ kcal&=kilocalories\hspace{.1 cm} available\\exer&=exercise\hspace{.1 cm}(DCCT\hspace{.1 cm} scale: \hspace{.1 cm}0-4))\\ FICD &= fetal,\hspace{.1 cm} infant,\hspace{.1 cm} childhood \hspace{.1 cm}distress\hspace{.1 cm}(surrogate\hspace{.1 cm} for\hspace{.1 cm} child\hspace{.1 cm} mortality)\\ p &= Air \hspace{.1 cm}pollution \hspace{.1 cm}caused \hspace{.1 cm}deaths\hspace{.1 cm}per\hspace{.1 cm}100,000\hspace{.1 cm}per\hspace{.1 cm}year\\ edu &= education\hspace{.1 cm} (years\hspace{.1 cm}in\hspace{.1 cm}school) \\gender&=sex(female=1, male=0)\\BMI&=body\hspace{.1 cm} mass\hspace{.1 cm} index(kg/m^2)) \end{align*}

\begin{align*} BMI&=0.61847 ((126.928 prot +136.42carbs - 196.14 fiber +477.144 TF-333.405PUFA)/kcal \\&\quad+ 0.09707 a -2.10094exer+0.77598 gender)- 0.01217FICD+ 0.06196 edu - 0.00832 p\end{align*}

(n = 336 cohorts, R^2 ^= 0.53)

FBS equation (derived from diabetes prevalence):

\begin{align*} prot &= protein\hspace{.1 cm} (g\hspace{.1 cm}per\hspace{.1 cm}day\hspace{.1 cm}available) \\ carbs &=carbohydrates \hspace{.1 cm} (g\hspace{.1 cm}per\hspace{.1 cm}day\hspace{.1 cm}available)\\fiber&=dietary\hspace{.1 cm} fiber\hspace{.1 cm} (g\hspace{.1 cm}per\hspace{.1 cm}day\hspace{.1 cm}available) \\ TF&=total \hspace{.1 cm}fat\hspace{.1 cm} (g\hspace{.1 cm}per\hspace{.1 cm}day\hspace{.1 cm}available) \\kcal&=kilocalories\hspace{.1 cm} available\\ exer &= exercise\hspace{.1 cm}(DCCT scale: 0-4)\\ t &= tobacco\hspace{.1 cm}(yes=1, no=0)\\p&=air\hspace{.1 cm}pollution\hspace{.1 cm}(deaths/100,000/year)\\FICD &= fetal,\hspace{.1 cm} infant,\hspace{.1 cm} childhood\hspace{.1 cm} distress\hspace{.1 cm} (surrogate\hspace{.1 cm}for\hspace{.1 cm}childhood\hspace{.1 cm}mortality)\\ edu &= education\hspace{.1 cm} (years\hspace{.1 cm}in\hspace{.1 cm}school) \\FBS&=fasting\hspace{.1 cm} blood\hspace{.1 cm} sugar \hspace{.1 cm}(simulated\hspace{.1 cm} from \hspace{.1 cm}diabetes\hspace{.1 cm} prevalence-IDF\hspace{.1 cm} data)\end{align*}

\begin{align*} FBS &= 0.61252(0.42603((36.8 prot + 19.528carbs - 28.07fiber + 55.98 TF)/kcal)- 0.28256 exer \\&\quad + 0.02488edu - 0.00090463p)- 0.00253FICD + 0.21252t \end{align*}

(n = 336 cohorts, R^2 ^= 0.50)

SBP formula:

\begin{align*} a &= alcohol \hspace{.1 cm}(\frac{g\hspace{.1 cm}consumed} {day})\\ K2 &= vitamin\hspace{.1 cm}K2\hspace{.1 cm} (\frac{\mu g\hspace{.08 cm}available}{day})\\kcal &= total\hspace{.1 cm}kcal\hspace{.1 cm}available\hspace{.1 cm}per\hspace{.1 cm}day\\t &= tobacco\hspace{.1 cm}(yes=1, no=0)\\ GDP &=percapita\hspace{.1 cm} gross\hspace{.1 cm}domestic\hspace{.1 cm}product\hspace{.1 cm}(USD)\\ FICD &=fetal,\hspace{.1 cm} infant,\hspace{.1 cm} childhood\hspace{.1 cm} distress \\p &= Air \hspace{.1 cm}pollution \hspace{.1 cm}caused \hspace{.1 cm}deaths\hspace{.1 cm}per\hspace{.1 cm}100,000\hspace{.1 cm}per\hspace{.1 cm}year\\gender &= sex\hspace{.1 cm} (female=1, male=0)\\SBP &= systolic\hspace{.1 cm} blood\hspace{.1 cm} pressure \hspace{.1 cm} (mm \hspace{.1 cm}Hg)\\SBP &= (0.78022 (0.66476(0.46343a - 530.96 (\frac{\ K2}{kcal})) +5.7996 t - 0.00004485 GDP + 0.02318FICD)\\&\quad -2.60763 gender \end{align*}

(n = 336 cohorts, R^2 ^= 0.43)

TC/HDL formula:

\begin{align*} a &= alcohol (g/day)\\ prot &= protein (g\hspace{.1 cm}per\hspace{.1 cm}day\hspace{.1 cm}available)\\kcal&=kilocalories\\CF&=carbohydrates (g) /fiber (g)\\TF&=total\hspace{.1 cm} fat \hspace{.1 cm}(g)\\PUFA &=polyunsaturated\hspace{.1 cm} fatty\hspace{.1 cm}acids\hspace{.1 cm} (g)\\ exer& = exercise\hspace{.1 cm}(DCCT \hspace{.1 cm}scale:\hspace{.1 cm} 0-4)\\GPD &=percapita\hspace{.1 cm} gross\hspace{.1 cm}domestic\hspace{.1 cm}product\hspace{.1 cm}(USD) \\FICD &=fetal,\hspace{.1 cm} infant,\hspace{.1 cm}childhood\hspace{.1 cm}distress\hspace{.1 cm}(surrogate\hspace{.1 cm}for\hspace{.1 cm}childhood\hspace{.1 cm}mortality)\\t&= tobacco\hspace{.1 cm}use (yes=1, no=0)\\gender&=sex\hspace{.1 cm}(female=1, male=0)\\TC/HDL &= total\hspace{.1 cm}cholesterol/HDL\hspace{.1 cm} ratio \end{align*}

\begin{align*} TC/HDL &= 0.39765 (0.93566 ((38.168 prot + 94.257TF- 173.817PUFA)/kcal + 0.03549a  \\&\quad+0.05667CF) -0.28628exer- 0.31859gender) + 0.00001843 GDP - 0.00702FICD\\&\quad+ 0.83485t \end{align*}

(n = 336 cohorts, R^2 ^= 0.82)

BMI and FBS were negatively correlated with early death from CVD (r = –0.17, *P *= 0.0020 and r = –0.06, *P *= 0.24, respectively) and did not enter the multiple regression formula for early death from CVD. However, in the wealthiest countries (per capita GDP > $15,000, n = 70 cohorts), BMI and FBS strongly correlated with early death from CVD (r = 0.54, *P *< 0.0001 and r = 0.53 *P < *0.0001, respectively). In these wealthy countries as opposed to all countries, TC/HDL correlated with increased CVD deaths. In the cohorts from the wealthiest countries, alcohol and vitamin K2 trended directly and inversely with early death from CVD ( r = 0.20, *P *= 0.10 and r = –0.15, *P *= 0.22, respectively). The formula for early CVD death in these countries was the following:

\begin{align*} a &= alcohol \hspace{.1 cm}(\frac{g\hspace{.1 cm}consumed} {day})\\ K2 &= vitamin\hspace{.1 cm}K2\hspace{.1 cm} (\frac{\mu g\hspace{.08 cm}available}{day})\\kcal &= total\hspace{.1 cm}kilocalories\hspace{.1 cm}available\hspace{.1 cm}per\hspace{.1 cm}day\\TC/HDL &= total\hspace{.1 cm}cholesterol / HDL-C\hspace{.1 cm}ratio\\ edu&=education\hspace{.1 cm} (years\hspace{.1 cm}in\hspace{.1 cm}school) \\GDP&=percapita\hspace{.1 cm}gross\hspace{.1 cm}domestic\hspace{.1 cm}product\hspace{.1 cm}(USD)\\t &= tobacco\hspace{.1 cm}(yes=1, no=0)\\ FICD &= fetal,\hspace{.1 cm}infant,\hspace{.1 cm}and \hspace{.1 cm}childhood\hspace{.1 cm}distress\\SBP &= systolic\hspace{.1 cm} blood\hspace{.1 cm} pressure \hspace{.1 cm} (mm \hspace{.1 cm}Hg)\\ BMI &= body\hspace{.1 cm}mass\hspace{.1 cm}index\hspace{.1 cm}(\frac{kg} {m2})\\ FBS &= fasting\hspace{.1 cm}blood\hspace{.1 cm}sugar\\gender &= sex\hspace{.1 cm}(female=1, male=0)\end{align*}

\begin{align*} EWC &= Early\hspace{.1 cm}death\hspace{.1 cm}by\hspace{.1 cm}CVD\hspace{.1 cm}(wealthy\hspace{.1 cm}countries:\hspace{.1 cm} GDP\hspace{.1 cm}>\hspace{.1 cm}\$ 15,000)\\ EWC &= 0.88264 (0.83008 (0.49627 (0.56947 (-25739\hspace{.1 cm}K2 /kcal + 17.097\hspace{.1 cm}a)+ 221.98\hspace{.1 cm}TC/HDL \\&\quad- 0.00789\hspace{.1 cm}GDP - 49.969\hspace{.1 cm}edu)+ 1171.6 t + 62.935\hspace{.1 cm}BMI+ 233.63\hspace{.1 cm}FBS \\&\quad+ 62.619\hspace{.1 cm}FICD) - 216.818\hspace{.1 cm}gender) + 10.591\hspace{.1 cm}SBP \end{align*}

   (n = 70 cohorts, R^2 ^= 0.68);

As described in the Methods, the multiple regression-derived formulae for early death from CVD (n = 168 countries and 336 cohorts) allows us to attribute proportions of risks to the variables in the formula in relationship to the overall 50% of the variance in risk (R^2 ^= 0.50) accounted for by the formula. The 35 wealthy countries had attributable CVD risks that differed markedly with the overall 168-country analysis. Table 13 contrasts the attributable risks of the overall analysis (n = 168 countries) and the 35 wealthy countries and also shows the attributable risks for each of the biometrics (BMI, FBS, SBP, and TC/HDL). Notably, the multiple regression formulae derived attributable risk quantification for the biometrics, except for SBP, included protein, carbohydrates/fiber, total fat/PUFA, and exercise, unlike the formulae for CVD risk. 

**Table 13:** Attributable risks for early death from CVD and attributable risks for associated risk factors* * (+) indicates the factor increases risk and (–) means the factor decreases the risk.

CVD Risk Factors	CVD Early Death (n=336 Cohorts)	CVD Early Death (n=70 Wealthy Cohorts)	BMI	FBS	SBP	TC/HDL
Alcohol	(+)0.38	(+)1.41	(+)0.29		(+)5.65	(+)7.52
Vitamin K2	(–)6.95	(–)0.77			(–)4.84	
Protein			(+)4.98	(+)4.99		(+)14.28
Carbohydrates/fiber			(+)2.16	(+)1.14		(+)4.65
Total fat/PUFA			(+)4.94	(+)1.62		(+)12.07
Exercise			(–)8.95	(–)9.05		(–)1.31
Tobacco use	(+)6.87	(+)2.78		(+)1.50	(+)3.04	(+)4.58
FBS		(+)10.22				
BMI		(+)9.89				
SBP	(+)9.01	(+)14.70				
TC/HDL		(+)1.03				
Gender (F=1, M=0)	(–)6.13	(–)12.42	(+)0.75		(–)14.39	(–)2.19
Air pollution	(+)9.15		(–)7.77	(–)6.56	(+)3.58	
FICD	(+)3.64	(+)9.46	(–)13.45	(–)16.61	(+)7.57	(–)18.2
GDP	(–)7.66	(–)2.77			(–)7.57	(+)16.91
Education		(–)2.19	(+)10.03	(+)8.19		
Total (%)	49.78	67.64	53.31	49.67	46.64	81.71

The big differences in the subset analyses of the wealthiest countries (n = 35, cohorts = 70) and the poor countries (n = 133, cohorts = 266) may be attributable to the factors in Table 14.

**Table 14:** Contrasting CVD risk factor data from wealthy and poor countries

#	CVD Risk Factors Mean Amounts in Wealthy (n=35) and Poor Countries (n=133)
1	Mean CVD deaths/100,000: wealthy—585/year, poor—1685/year
2	Mean kcal available per day: wealthy—3224, poor—2523
3	Mean animal product intake per 2000 kcal/day: wealthy—585, poor—303
4	Mean sugar intake per 2000 kcal/day: wealthy—241, poor—192
5	Mean vitamin K2 per 2000 kcal: wealthy—27.2 µg/day, poor—10.4 µg/day
6	Mean tobacco use: wealthy countries—23%, poor countries—19%
7	Mean BMI: wealthy—26.6 kg/m^2^, poor—25.3 kg/m^2^
8	Mean diabetes prevalence (40-49 years old): wealthy—21.9%, poor—16.5%
9	Mean SBP: wealthy—122.4 mm Hg, poor—127.5 mm Hg
10	Mean early childhood mortality per 100,000: wealthy—4.86/year, poor—50.1/year
11	Mean serum cholesterol: wealthy—5.24 mmol, poor—4.57

All the data used for this analysis is available for viewing and downloading on the website of DKC (see Appendix 1).

## Discussion

When the analysis was limited only to wealthy countries (per capita GDP > $15,000, n = 70 cohorts), BMI, FBS, SBP, TC/HDL, tobacco, early childhood mortality, GDP, and education entered the multiple regression formula while exercise and macronutrients other than alcohol did not. This shows that the data from developing countries is essential for an evidence-based analysis of diet and other risk factors for early death from CVD and for analyzing the risk factors for the associated biometrics. All the biometrics entered the formula for early CVD death, and exercise and macronutrients (i.e. protein, carbs/fiber, and total fat/PUFA) are prominent in the multiple regression formula for BMI, FBS, and TC/HDL. Consequently, further analysis could guide to a more evidence-based and fuller appreciation of the roles of exercise and diet in the pathogenesis of CVD, especially in wealthy countries. Inadequate vitamin K2 intake was the dominant dietary problem in poor countries. The complete lack of a statistical effect of vitamin K1 on early CVD deaths (r = –0.04 *P *= 0.45) or SBP (r =  –0.01 *P *= 0.93) further supports the hypothesis that the molecular differences between phylloquinone (K1) and menaquinones (K2) account for why vitamin K-dependent proteins in arterial walls require vitamin K2 and not vitamin K1 to prevent the deposition of calcium and the stiffening of arterial walls.

Wealthy countries tended to have more dietary animal products, sugar, and vitamin K2 along with higher mean BMIs and FBSs, while cohorts in poor countries had higher mean SBPs. As people in developing countries have adopted the diets and lifestyles of those in Western countries, their premature deaths from CVD have declined, but, like people in wealthy countries, they have become increasingly susceptible to obesity and type 2 diabetes [29].

In the 168-country analysis and the 35-wealthy-country analysis, alcohol trended with early CVD (r = 0.10, *P* = 0.08 and r = 0.20, *P* = 0.10, respectively) despite correlating positively with vitamin K2 µg/kcal/day (r = 0.33,* P* < 0.0001). The correlations of alcohol with tobacco use (r = 0.47, *P* < 0.0001) and SBP (r = 0.32, *P* < 0.0001) could also have affected the association of alcohol with CVD death. Notably, alcohol was the dominant dietary risk factor related to SBP, followed by the inverse correlation of SBP with vitamin K2. Vitamin K2's role in keeping calcium out of arteries and into bones may account for its beneficial role in blood pressure as well as early CVD deaths.

The World Heart Federation (WHF) attributed five percent of CVD risk to insufficient physical activity (i.e. the less the physical activity the more the early CVD deaths). However, the current study found that exercise correlated positively with early CVD death (i.e. the more the physical activity the more the early CVD deaths) in the univariate analysis (exercise: r = 0.35, *P* < 0.0001). Because of the association of exercise with other CVD risk factors (e.g. vitamin K2 : r = –0.30,* P* < 0.0001), it did not appear in the formulae modeling early death from CVD or with SBP. As might be expected, exercise did correlate negatively in the formulae modeling BMI, FBS, and TC/HDL and in the attributable risk for those biometric variables (Table 13). In the FAO/WHO/IHME/IDF participants, the potential beneficial effects of exercise on preventing obesity, type 2 diabetes, SBP, and CVD could be underestimated because of the low physical activity levels, averaging only slightly more than 30 minutes of moderate-intensity aerobic exercise five days per week.

The attributable risks of some CVD risk factors roughly accorded with the analyses of the WHF and the WHO: tobacco use 6.87% versus WHF = 9% [30], high blood pressure = 9.01% versus WHF = 13% [29], and air pollution = 9.15% versus WHO = 28% [22].

Limitations of this FAO/WHO/IDF/IHME analysis include: (1) we had access to per capita food availability rather than food consumption, except for alcohol, (2) we did not assess changes in physical activity, tobacco use, and other variables over decades, (3) we imputed missing data regarding physical activity, tobacco use, early childhood mortality, and years of education, (4) data on MK-4 from 547 selected items from the USDA nutrition database may not have been entirely representative of the MK-4 levels in the USA or other countries, (5) data on long chain menaquinone (MK-5–MK13) were limited to a few European countries and Japan.

## Conclusions

These multiple-regression equations relating CVD-associated outcomes to diet and other risk factors should be confirmed with prospective studies on individuals.

Because of these findings, health regulatory agencies of countries should more inclusively measure vitamin K2 levels (MK-4–MK-13) in foods and should designate levels for adequate intakes (AIs) for vitamin K2. In wealthy countries, given the health concerns with excessive meat, dairy, and eggs (e.g. obesity, type 2 diabetes, and cancer), vitamin K2 should be optimally boosted from fermented plants containing long chain menaquinones rather than from animal products. Likewise, for people in poor countries, increasing the intake of fermented foods such as sauerkraut, miso, and natto would be the most cost-effective and healthful way to boost vitamin K2 to adequate levels.

## Appendices

### The source data for this analysis

**Supplementary Table 1**. **Plant-based food groups—kcal/day available per capita in worldwide countries**: http://whistleblowerdoctor.org/2016/05/supplementary-table-1-plant-based-food-groups-kcalsday-available-percapita/

**Supplementary Table 2. Animal-based food groups—kcal/day available per capita in worldwide countries: **http://whistleblowerdoctor.org/2016/05/supplementary-table-2-animal-based-food-groups-kcalsday-available-per-capita/

**Supplementary Table 3. Template for conversions of food group data (per 100 g portion) into nutrient profiles: **http://whistleblowerdoctor.org/2016/05/supplementary-table-3-template/

**Supplementary Table 4. Macronutrients available in countries: **http://whistleblowerdoctor.org/2016/05/supplementary-table-4-macronutrients-available-in-countries/

**Supplementary Table 5. Trans fatty acids (g per capita/day available), alcohol consumed g/day (female and male) and vitamin K1 and vitamin K2 (µg/day per capita available):** http://whistleblowerdoctor.org/2016/05/supplementary-table-5-more-nutrients/

**Supplementary Table 6**. **Formulas for imputing absent values for variables.**http://whistleblowerdoctor.org/2016/05/supplementary-table-6-formulas-for-imputing-absent-variables/

**Excel worksheets for nutrient profiles of food commodities**: http://whistleblowerdoctor.org/2016/05/worksheets-for-nutrient-profiles-of-food-commodities/

**Food commodity to macronutrient Excel sheet**: http://whistleblowerdoctor.org/wp-content/uploads/2016/05/Food-commodity-to-MN-excelsheet.xls

**CVD Diet Risks (downloadable source data for diet-related risk factors): **http://whistleblowerdoctor.org/wp-content/uploads/2016/05/CVDDietRisks.xls

**CVD Non Diet Risks (downloadable source data for CVD early deaths and non-dietary risk factors for CVD)**: http://whistleblowerdoctor.org/wp-content/uploads/2016/05/CVDNonDietRisks.xls

**IHME_2013_DEATHS ages 15-64 from CVD female:** http://whistleblowerdoctor.org/wp-content/uploads/2016/05/IHME_GBD_2013_DEATHS_1990_2013_CVD-female.pdf

**IHME_2013_DEATHS ages 15-64 from CVD male:** http://whistleblowerdoctor.org/wp-content/uploads/2016/05/IHME_GBD_2013_DEATHS_1990_2013_CVD-male.pdf

**Attributable risks Excel worksheet: **http://whistleblowerdoctor.org/wp-content/uploads/2016/05/attributable-risks.xlsx

**SAS calculations code**: http://whistleblowerdoctor.org/wp-content/uploads/2016/05/SAS-calculations-code.pdf

**SAS source code: **http://whistleblowerdoctor.org/wp-content/uploads/2016/05/SAS-source-code.pdf
